# Cerebrospinal Fluid Density Measured Using Hounsfield Units on a Head CT Scan May Be Utilized as a Radiologic Indicator of Shunt Malfunction: A Pilot Study

**DOI:** 10.7759/cureus.87154

**Published:** 2025-07-02

**Authors:** Juan Vigo-Prieto, Juan Vicenty, Orlando De Jesus, Ashlie Maldonado-Pérez, Gloria Carrasquillo, Roberto Davila Martinez

**Affiliations:** 1 Neurological Surgery, University of Puerto Rico, Medical Sciences Campus, San Juan, PRI; 2 Neurosurgery, University of Puerto Rico, Medical Sciences Campus, San Juan, PRI; 3 Graduate Medical Education, School of Health Professions, University of Puerto Rico, Medical Sciences Campus, San Juan, PRI

**Keywords:** cerebrospinal fluid, ct scan, hounsfield unit, hydrocephalus, shunt malfunction, ventricular shunt

## Abstract

Objective: For patients of all ages, symptoms of headache, nausea, vomiting, or altered mental status due to shunt malfunction are frequent causes of visits to the emergency department. However, in shunted patients, those symptoms can also be attributed to other conditions. In this study, we evaluated the use of cerebrospinal fluid density measurements on the head CT scans as a potential radiologic indicator of shunt malfunction. The study aimed to evaluate whether lower ventricular cerebrospinal fluid Hounsfield unit values on pre-operative CT scans correlate with shunt malfunction, as reflected by an increase in Hounsfield unit following revision.

Methods: The University of Puerto Rico Neurosurgery Database was scrutinized to identify patients diagnosed with hydrocephalus who underwent a ventricular shunt revision from August 2021 to August 2024. For each patient, we measured the mean ventricular cerebrospinal fluid Hounsfield unit value within the atrium of the lateral ventricles on the CT scan axial view before and after the shunt revision. The correlation between the preoperative group and the postoperative group was assessed using the non-parametric Spearman’s rank correlation coefficient.

Results: The cohort consisted of 34 patients, aged one to 71 years, with a median age of 35.5 years (interquartile range, 26-45 years). Eighteen percent of the cohort was under 18 years of age. There were 17 females and 17 males. The mean ventricular cerebrospinal fluid pre-operative Hounsfield unit value was 5.2 (range: -1 to 12), and the postoperative mean Hounsfield unit value was 6.0 (range: -2 to 13). The difference in Hounsfield unit values between the preoperative and postoperative patients with shunt malfunction who underwent shunt revision was significant (p-value = 0.045).

Conclusion: Our study findings suggest that lower ventricular cerebrospinal fluid Hounsfield unit values may correlate with shunt malfunction. When a prior head CT scan is available, Hounsfield unit comparison can serve as an adjunctive diagnostic tool to guide clinical decision-making.

## Introduction

Among the pediatric worldwide population, it is estimated that more than 400,000 new patients are diagnosed annually with hydrocephalus [1]. In the United States, the management of pediatric hydrocephalus leads to approximately 39,000 hospitalizations and 36,000 shunt surgeries annually [2,3]. Hydrocephalus, particularly in the setting of shunt malfunction, necessitates an accurate and timely diagnosis. Clinical parameters, including headache, nausea, vomiting, irritability, and altered mental status, have been described as predictors for shunt malfunction [4,5]. However, these symptoms can also be present in other conditions. In patients with a ventricular shunt, symptoms of shunt malfunction can create a diagnostic dilemma regarding whether the system is functioning adequately or if the patient requires a revision of the shunt. Specific changes detected in brain imaging, such as increased ventricle size, stretching or distortion of the septum pellucidum, or effacement of pericerebral spaces, are generally used as indicators for possible shunt malfunction and, thus, the need for a revision [5]. However, imaging changes can be an unreliable indicator of shunt malfunction in a selected group of patients, as the ventricle size may not increase. Studies have demonstrated that a head CT scan has a sensitivity ranging from 53% to 92% and a specificity between 76% and 93% for detecting ventricular shunt malfunction [6,7]. Albugami et al. evaluated the preoperative ventricular size of 42 patients who underwent ventricular shunt revision, demonstrating that up to 43% of patients presented with minimal or no ventricular enlargement [8]. New methods are needed to predict shunt malfunction and help physicians decide whether a shunt revision is necessary.

Modern radiological imaging provides valuable techniques for measuring cerebrospinal fluid (CSF) flow through the shunt catheter. Several authors have utilized MRI phase contrast images to measure the CSF flow within the shunt catheter [9-13]. Although this CSF flow measurement in shunted patients is noninvasive and can be used to assess adequate CSF flow in patients with suspected shunt malfunction, it is time-consuming and challenging to perform during emergent situations. The CT scanner provides a valuable tool for measuring the characteristics of human body tissue using attenuation values, which calculate the amount of X-ray energy absorbed by the tissue. These measurements are converted to a linear unit scale named the Hounsfield unit (HU), in which distilled water is arbitrarily assigned a value of 0, to which the values of other tissues are compared. Tissue HU measurements have been paramount in surgical and non-surgical medicine. Some authors have used spine HU measurements on routine CT scans to estimate bone mineral density in patients with osteoporosis and other musculoskeletal conditions. The results are comparable to other traditional tests measuring bone mineral density [14-16]. This approach for determining bone density in the spine has been validated [17-19]. Similarly, HU measurements have been used as a simple, non-invasive tool for the early prediction of aseptic bone necrosis following autologous cranioplasty [20].

Patients with shunt malfunction develop elevated intracranial pressure (ICP) when a sufficient amount of CSF is not drained. Boulton et al. demonstrated that elevated ICP produces a higher CSF transport through the lymphatic and arachnoid villi routes [21]. Based on their conclusions, we suspected that patients with shunt malfunction may have a lower CSF protein content due to a higher efflux of the proteins. Consequently, in these patients, preoperative CSF HU measurements may be lower than postoperative ones because the CSF density could have decreased due to the lowered CSF protein content. We hypothesized that patients with shunt malfunction might have lower CSF HU measurements on the preoperative CT scan compared to the postoperative head CT scan after the shunt is revised. If the hypothesis is verified, the head CT scan of a patient presenting symptoms of shunt malfunction can be compared with a prior baseline study performed when no symptoms were present, to suggest shunt malfunction. For this study, preoperative head CT scans were compared with postoperative ones in patients with shunt malfunction who underwent a shunt revision. We assessed the use of CSF density measurements on the head CT scans as a potential radiologic indicator of shunt malfunction. The purpose of this study was to evaluate whether lower ventricular CSF HU values on pre-operative CT scans correlate with shunt malfunction, as reflected by an increase in HU following revision. To our knowledge, no prior study has quantitatively assessed HU differences in the ventricular CSF to evaluate for the presence of shunt malfunction.

## Materials and methods

**Figure 1:**
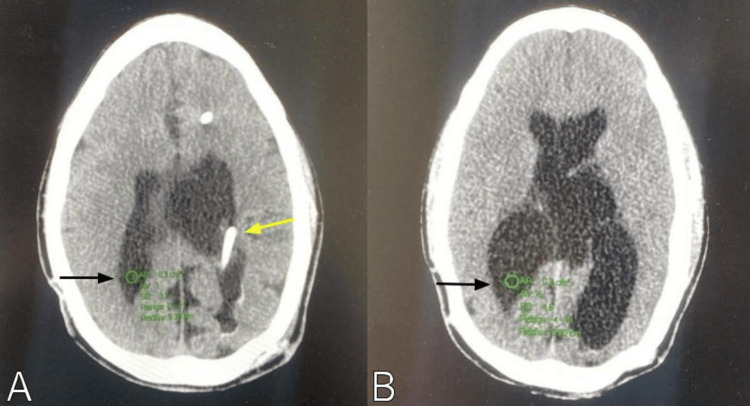
Axial head CT scan images showing the 0.3 cm radius region of interest circle at the patient's lateral ventricle atrium (black arrow). (A) Measurement is done at the ventricle contralateral to the shunt catheter, which is marked by the yellow arrow. (B) The circle is placed in an area without choroid plexus.

A retrospective study was performed using the University of Puerto Rico Neurosurgery Database to identify patients diagnosed with hydrocephalus who underwent a ventricular shunt revision due to shunt malfunction from August 2021 to August 2024. Patients with a diagnosis of normal pressure hydrocephalus (NPH), post-subarachnoid hemorrhage hydrocephalus, over-shunting, and shunt malfunction due to an abdominal pseudocyst were excluded to ensure the homogeneity of the sample. Patients with no preoperative imaging available in the radiology archives were also excluded. Using the axial head CT scan image, measurements of the mean HU at the patient's lateral ventricle atrium at a region of interest (ROI) circle of 0.3 cm^2^ with a 0.3 cm radius were done (Figure 1).

The picture archiving and communication system (PACS) used was the Vue PACS (Phillips Medical Systems, The Netherlands). The same author performed all HU measurements to prevent interobserver variability. To avoid confounding results due to the hyperintense signal of the ventricular shunt catheter, we measured CSF HU in the contralateral ventricle. CT scan HU measurements were acquired for each patient before and after the shunt revision procedure. The same scanner was used throughout the study’s inclusion period, thereby avoiding inter-scanner variability. The CT study acquisition protocol used was uniform among all patients, as the examinations were performed using a similar shunt malfunction protocol. 

Descriptive statistics were used to report frequency, percentage, and mean values. Continuous variables with a non-normal distribution were reported as median with interquartile range (IQR) from the 25th to 75th percentiles. The statistical analyses were conducted in Python 3.x (NumPy, SciPy, and Matplotlib), using SciPy’s Spearman function to obtain Spearman’s correlation coefficient and its significance. The correlation between the preoperative HU value group and the postoperative HU value group was assessed using the non-parametric Spearman’s rank correlation coefficient. A significance level of p < 0.05 was used. The study was approved by the Institutional Review Board of the University of Puerto Rico Medical Sciences Campus (protocol # 2408271821).

## Results

The cohort consisted of 34 patients aged one to 71 years. The cohort's median age was 35.5 years (IQR 26-45 years). Eighteen percent of the cohort was under 18 years old. There were 17 females and 17 males. The mean preoperative HU value was 5.2 (range -1 to 12), and the postoperative mean HU was 6.0 (range -2 to 13) (Table 1).

**Table 1:** Cerebrospinal fluid Hounsfield unit (HU) values for the 34 patients before and after shunt revision

Patient #	Preoperative HU	Postoperative HU
1	5	6
2	2	5
3	4	2
4	5	6
5	3	4
6	3	4
7	9	12
8	6	3
9	8	1
10	5	1
11	4	5
12	1	8
13	5	5
14	4	6
15	5	12
16	7	7
17	6	2
18	6	7
19	5	7
20	5	12
21	4	4
22	10	12
23	4	9
24	6	7
25	6	7
26	6	9
27	3	-2
28	10	7
29	12	13
30	8	3
31	2	6
32	1	3
33	7	6
34	-1	4

**Figure 2:**
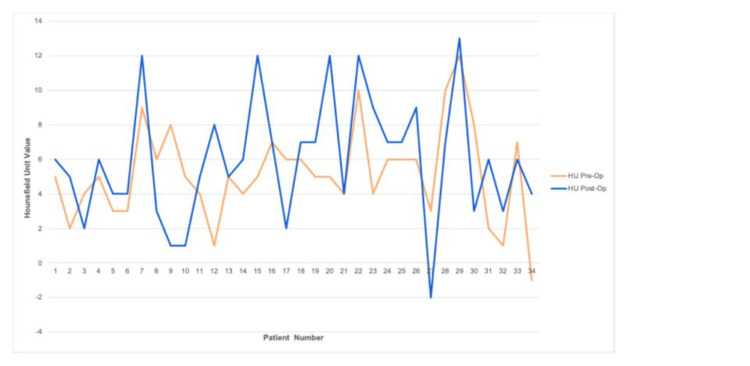
Line plot illustrating the head CT scan cerebrospinal Hounsfield unit values for each of the 34 patients before and after the shunt revision. The yellow line depicts the preoperative HU values. The blue line depicts the postoperative HU values. HU = Hounsfield unit

**Figure 3:**
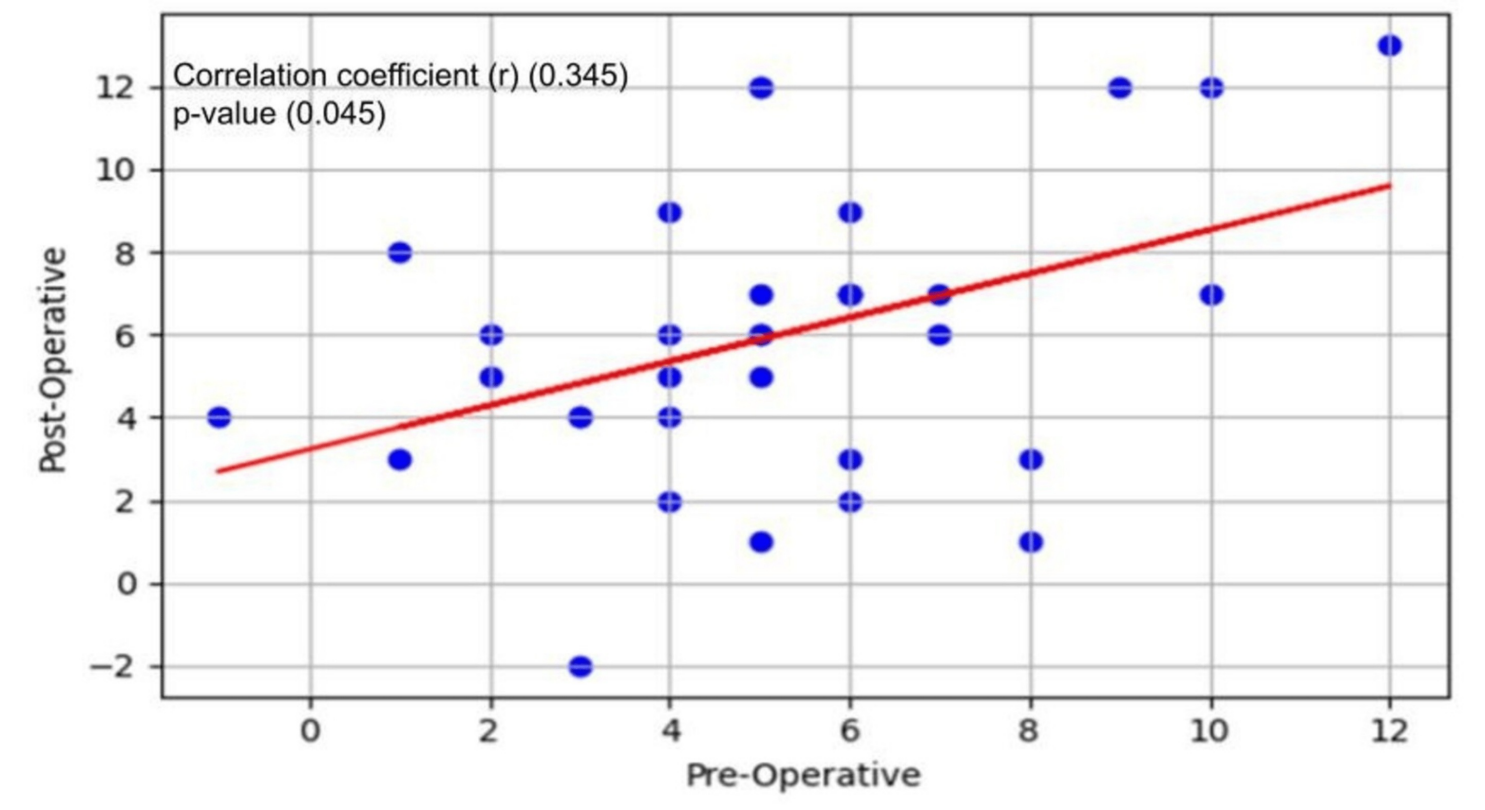
Spearman’s correlation plot illustrates the relationship between preoperative and postoperative head CT scan cerebrospinal Hounsfield unit values. Each blue dot in the scatter plot represents a pair of preoperative and postoperative values. The red line represents the best-fit regression line illustrating the trend of the data.

Figure 2 depicts a line plot illustration of each patient's head CT scan HU value before and after the shunt revision. Using Spearman’s correlation, the correlation coefficient between both groups was 0.345 (Figure 3). The difference between the preoperative HU group and the postoperative HU group values was significant, with a p-value = 0.045.

## Discussion

For the cohort of patients with suspected shunt malfunction, we assumed a difference in the head CT scan CSF HU measurements before and after a shunt revision. We hypothesized that the CSF HU value would be lower in the preoperative head CT scan than in the postoperative scan due to decreased CSF protein levels as a result of the elevated ICP secondary to shunt malfunction. Our study found a significant difference in the preoperative head CT scan ventricular CSF HU values of patients with shunt malfunction compared to the values in the postoperative CT scan. Based on our findings, the CT scan ventricular CSF HU value may be used as an objective guide to suggest shunt malfunction.

Several authors have documented decreased CSF protein levels in patients with elevated ICP. Berezovsky et al. demonstrated that in patients with idiopathic intracranial hypertension (IIH), there is a significant decrease in CSF protein for each 1 cm H_2_O increase in CSF opening pressure [22]. Wilhelmy et al. found significantly decreased CSF protein levels in patients with aqueductal stenosis and IIH compared with controls; however, in patients with communicating hydrocephalus or NPH, the protein levels were not changed compared with those of the controls [23]. In patients with communicating hydrocephalus or NPH, there is no flow disruption within the ventricles, and the ICP is not elevated in NPH [23]. Wilhelmy et al. suggested that the reduced overall CSF protein concentrations in patients with aqueductal stenosis and IIH may be a counterregulatory mechanism aimed at reducing intracranial CSF volume and ICP [23]. Vinje et al. demonstrated that elevations in ICP affect pressure-driven flow due to arterial pulsations and, simultaneously, impact CSF clearance [24].

The intraluminal density of an obstructed proximal shunt catheter had been previously investigated to determine if it can be used to indicate catheter obstruction. Apsara et al. measured the intraluminal HU value of the proximal shunt catheter in pediatric patients with a documented proximal shunt malfunction [25]. They postulated that debris within the obstructed proximal catheter would increase the intraluminal density; however, they did not identify a difference in the intraluminal HU values before and after the revision [25]. For our study, we measured the ventricular CSF density instead of the intraluminal proximal shunt catheter density.

Our study contains several limitations. Many patients who underwent shunt revision during the study period had to be excluded because the preoperative CT scan was performed at the referring hospital and was unavailable for review, thereby significantly reducing the sample size. A multi-institutional research study could substantially increase the sample size and could be used to confirm or contradict our findings. Our study results require a larger study for validation. In addition, the study did not incorporate interrater or intra-rater reliability as assessments. The time interval between preoperative and postoperative imaging varied among patients; however, all postoperative images were obtained within 48 hours following the procedure. The study did not include a control group, as no intervention would have been performed in patients who did not undergo shunt revision. Furthermore, measurements of the mean HU in an axial plane ROI can induce errors because the measure is acquired with only two dimensions, and the values obtained can be underestimated or overestimated. This error could be minimized if the volume of interest is measured instead of an axial ROI, as this calculates the CSF HU in a 3D area. Unfortunately, the computer software used did not allow us to perform ROI volumetric measurements for our study. The study did not analyze the preoperative CSF protein level, as no comparison with a postoperative sample could have been performed due to unavailability. Our study cohort consisted exclusively of patients who had undergone a prior shunt placement and did not include new patients who had a shunt placed for the first time. Therefore, the impact of this study on patients with a new shunt remains to be evaluated. In the future, new shunted patients can provide a cohort where the CSF HU values can be compared before and after the shunt procedure.

## Conclusions

Our study findings suggest that lower ventricular CSF HU values may correlate with shunt malfunction. When a prior head CT scan is available, HU comparison can serve as an adjunctive diagnostic tool to guide clinical decision-making. Further studies should be conducted to investigate other characteristics of the CSF in these patients, such as protein content, highlighting the importance of this research.
